# Prediction of the Time-Dependent Elastic Modulus of Fly-Ash Concrete Under Sustained Loads

**DOI:** 10.3390/ma19030559

**Published:** 2026-01-30

**Authors:** Zhuoran Chen, Minghui Liu, Yurong Zhang, Siyi Jia

**Affiliations:** 1 School of Civil Engineering, Beijing Jiaotong University, Beijing 100044, China; 2 College of Civil Engineering, Zhejiang University of Technology, Hangzhou 310014, China; 3 Laboratory of Green Construction and Intelligent Operation & Maintenance for Coastal Infrastructure, Zhejiang University of Technology, Hangzhou 310014, China; 4 Faculty of Science and Engineering, Global Center for Science and Engineering, Waseda University, Tokyo 169-8555, Japan; syjia@aoni.waseda.jp

**Keywords:** fly-ash concrete, elastic modulus, sustained load, time-dependent properties

## Abstract

In this paper, the time-dependent properties of the elastic modulus of fly ash concrete under sustained compressive load were studied. An experiment was conducted and showed an increment of elastic modulus for two types of fly ash concrete (20% and 40% fly ash replacement) under sustained load. The mechanisms of this increment were analyzed, and two Representative Volume Elements (RVEs) were established to represent the micro-heterogeneous space of binder and concrete based on continuum mechanics. The shrinking core models of hydration and pozzolanic reaction were adopted to quantify the volume fraction of each phase within the binder RVE. A prediction model was proposed by incorporating the effects of extra hydration and time-dependent aggregate concentration rate under sustained load. Finally, parameter analysis including the influences of initial loading age and the loading level was conducted.

## 1. Introduction

Sustained load, when under a certain degree, can increase the strength and the elasticity of the loaded concrete. This concept was first raised by Chatelier over a century ago, who suggested that the external loads accelerate the hydration of cement [1]. Experiments were later carried out to corroborate this idea; experimental data have proved a strength increase of loaded concrete up to 15% higher than their load-free control group [2,3,4,5,6,7,8]. The strength and elasticity data in some creep tests have further verified this extra gain [9,10,11]. In Coutinho’s work, samples of loaded concrete were submitted to thermogravimetric analysis, and results confirmed the fact that there is a greater amount of free calcium hydroxide in loaded samples [10].

However, the incorporation of supplementary cementitious materials (SCMs) and waste/by-products for sustainability introduces additional complexity to the time-dependent behavior of concrete. Recent studies have demonstrated that materials such as metakaolin combined with laterite rock aggregates can significantly enhance mechanical performance through pore refinement and interfacial transition zone (ITZ) densification. Similarly, the synergistic use of waste glass cullet and snail shell powder has been proven to improve long-term strength via pozzolanic activity and filler effects [12,13]. These findings highlight that the microstructural evolution in eco-friendly concrete is governed by complex reaction kinetics.

In the realm of predictive modeling, empirical approaches such as the model by Han et al. have attempted to incorporate sustained load effects into strength development [14]. More recently, machine learning (ML) and numerical approaches have achieved significant success in predicting the elastic modulus of complex materials, including cellular structures and ultra-high-performance concrete (UHPC) [15,16]. Nevertheless, a critical limitation of these data-driven models is their reliance on short-term or quasi-static test data. Consequently, while effective for capturing instantaneous responses, they fail to account for the time-dependent evolution of stiffness specifically induced by sustained loading conditions.

Quantification for the time-dependent elastic modulus of loaded concrete becomes more complicated when fly-ash or silica fume is added to the mixture [17]; it requires the knowledge of the mechanism of sustained load and the microstructures of the heterogeneous material, which limits the application of empirical strength or elastic modulus models on this subject. To address this, this paper adopts the framework of continuum mechanics. Ulm has contributed to the prediction of concrete’s stiffness evolution by attributing it to the elasticity of the non-aging phases (clinker, hydrate, air, and water) and their volume fractions [18,19,20]. Moreover, time-dependent reaction kinetics of hydration and pozzolanic reaction need to be considered, so the shrinking core model is selected here for its applicability and capability of manifesting the mutual effect between cement hydration and pozzolanic reaction [21,22].

Nevertheless, the specific application of these theories to loaded fly-ash concrete still faces the following challenges: First, existing elastic modulus models generally neglect sustained-load-induced microstructural evolution. Second, fly-ash systems introduce time-dependent hydration and pozzolanic kinetics that make empirical models unreliable. Third, there is a lack of mechanism-based, micromechanical models validated by long-term experiments for fly-ash concrete under sustained load.

To bridge these gaps, this study establishes a two-level Representative Volume Element (RVE) micromechanical framework specifically designed for fly-ash concrete under sustained load. Distinct from previous approaches, this framework explicitly incorporates load-accelerated hydration and pozzolanic reaction kinetics to accurately capture microstructural densification. The theoretical predictions are rigorously validated through experimental verification of time-dependent elastic modulus enhancement across varying fly-ash replacement levels. Finally, the influence of initial loading age and stress levels on elasticity development is systematically investigated.

## 2. Materials and Methods

### 2.1. Theoretical Framework

#### 2.1.1. Discussion of the Mechanism

As mentioned above, many experiments have confirmed the fact that applying external load to concrete can enhance its mechanical properties. Some explanations attributed this phenomenon to the extra hydration of cement under sustained load [5,10]. Solubility of clinkers is enhanced under sustained load, producing a stiffer gel structure [1,10]. After the setting of concrete, hydrate solids in the cement paste become so stiff that macroscopic stresses barely change the pore structure; therefore, the pressure of both capillary and absorbed water remains unaffected by macroscopic stresses [23]. As a result, the macroscopic stresses on the solubility of clinkers should be non-hydrostatic [24]; for solid surfaces normal to the applied load, the solubility enhancement of the solid framework can be quantified as [25]:

(1) $$\frac{\gamma_{a}c_{a}}{\gamma_{p}c_{p}}=\exp\frac{v_{0}}{RT}[(a-p)(1-\frac{a+2p}{3K})+\frac{1}{2}\frac{{\left(a-p\right)}^{2}}{E}]$$

where $\gamma_{p},c_{p}$ are reaction and concentration rate of solid at hydrostatic pressure p, $\gamma_{a},c_{a}$ are reaction and concentration rate of solid at non-hydrostatic conditions with uniaxial stress, a, $v_{0}$ is molar volume of dissolved solid, K,E are the bulk modulus and Young’s modulus of solid, respectively, R is the universal gas constant, and T is the absolute temperature.

Concentration rate of aggregate is another key factor in determining the mechanical properties of concrete. Specifically, the elasticity of concrete is mainly controlled by the elasticity and concentration of the aggregate. The volume of hydration products is smaller than that of clinkers and water; this means a higher hydration degree leads to a higher concentration rate of aggregate, which can also be taken as a cause for the extra gain of strength in loaded concrete.

#### 2.1.2. Micromechanical Representation of Fly-Ash Concrete

**Figure 1:**
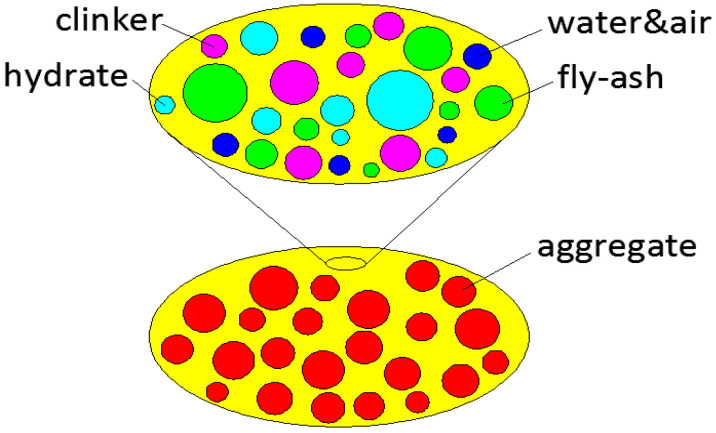
Upscaling of RVE.

In continuum mechanics, a Representative Volume Element (RVE) is defined as a micro-heterogeneous space with its characteristic size significantly larger than that of its composing phases [20]. In this study, two different sizes of RVE were established to represent the micro-heterogeneous space of binder and concrete. Quantification of elasticity needs the upscaling from the smaller RVE to the larger one (see Figure 1).

Determining the mechanical properties of a heterogeneous RVE needs the following: 1. identification of composing phases; 2. physical attributes of each phase; 3. volume fraction of each phase.

In our model, the RVE for the binder consists of cement clinkers, fly-ash particles, hydrates, water, and air. Considering an unsealed condition, the bulk and shear modulus of water are expected to be zero; then, further simplification was undertaken to combine water and air as one single phase. Representative particles of all phases are assumed to be spherical. RVE for the binder-aggregate dimension is a matrix-inclusion type that consists of spherical aggregate inclusions and binder matrix.

Describing the elasticity development of fly-ash concrete requires the knowledge of hydration and pozzolanic reaction degree at any given time to quantify the volume fraction of each phase. The shrinking core hydration model was selected, and its differential equation for cement hydration is shown as Equations (2) and (3) [21].

(2) $$\frac{da_{i}}{dt}=\frac{3C_{W\infty }}{r_{0}\rho_{c}(v+w_{g})}\times \frac{1}{(\frac{1}{k_{d}}-\frac{r_{0}}{D_{e}})+\frac{r_{0}{\left(1-a\right)}^{-\frac{1}{3}}}{D_{e}}+\frac{1}{k_{r}}{\left(1-a\right)}^{-\frac{2}{3}}}$$

(3) $$a=\frac{\sum_{i=1}^{4}a_{i}c_{i}}{\sum_{i=1}^{4}c_{i}}$$

where a is the weighted average reaction degree of cement, which can be calculated from the degree for the compounds of cement $a_{i}$ (C_3_S, C_2_S, C_3_A and C_4_AF); $\rho_{c}$ is the density of cement; v is the mass stoichiometric ratio of water to cement; $w_{g}$ mass ratio of physically bound water in cement paste; $r_{0}$ is the uniform cement particle radius that is related to the Blaine fineness S of cement and $\rho_{c}$; $C_{w\infty }$ is the amount of water in capillary pores; $k_{r}$ is the coefficient of reaction rate per unit area of the reaction interface.

$k_{d}$, $D_{e}$, $k_{r}$ are three parameters controlling three distinct stages in the hydration process. $k_{d}$ is the mass transfer coefficient at early stages of hydration and assumed to be a function of hydration rate and two constants, B and C.

(4) $$k_{d}=\frac{B}{a^{1.5}}+Ca^{3}$$

$D_{e}$ is the effective diffusion coefficient of water and is also assumed to be a function of hydration rate and a constant $D_{e0}$. $D_{e0}$ is the initial effective diffusion coefficient of water through the hydrate layer.

(5) $$D_{e}=D_{e0}\ln(\frac{1}{a})$$

For the similarities of reaction mechanisms between hydration and pozzolanic reaction, the differential equation for the pozzolanic reaction can be expressed as

(6) $$\frac{da_{F}}{dt}=\frac{m_{CH}(t)}{P}\times \frac{3\rho_{W}}{v_{Fa}r_{F}\rho_{F}}\times \frac{1}{(\frac{1}{k_{Fd}}-\frac{r_{0}}{D_{Fe}})+\frac{r_{0}{\left(1-a_{F}\right)}^{-\frac{1}{3}}}{D_{Fe}}+\frac{1}{k_{Fr}}{\left(1-a_{F}\right)}^{-\frac{2}{3}}}$$

where $r_{0}$ is initial uniform radius of the fly-ash particles; $a_{F}$ is the reaction rate of fly-ash particles, P is the mass of fly-ash, $\rho_{W},\rho_{F}$ are the densities of water and fly-ash, $r_{F}$ is the uniform radius of fly-ash particles, $v_{Fa}$ is the equivalent mass ratio of fly-ash to calcium hydroxide; $k_{Fd}$ is the reaction rate constant controlling the diffusion-controlled stage of the pozzolanic reaction; $k_{Fr}$ is the reaction rate constant controlling the phase-boundary reaction stage of the pozzolanic reaction. The mass of calcium hydroxide at time t $m_{ch}(t)$ writes

(7) $$m_{ch}(t)=RCH\times C_{0}\times a-v_{FA}a_{FA}P$$

where RCH is the mass of calcium hydroxide produced from 1 g cement. C0 is the mass of water in the mixture.

In loaded concrete, the reaction rate of cement hydration and pozzolanic reaction is accelerated

(8) $$\frac{da^{’}}{dt}=\varepsilon_{C}\frac{da}{dt}$$

(9) $$\frac{d{a_{F}}^{’}}{dt}=\varepsilon_{F}\frac{da_{F}}{dt}$$

$\varepsilon_{C}$ and $\varepsilon_{F}$ are the acceleration factors for cement hydration and pozzolanic reaction. Applied loads are normal to all reacting surfaces for spherical phases. Cooperating Equation (1), $\varepsilon_{C}$ and $\varepsilon_{F}$ can be derived as follows

(10) $$\varepsilon_{c}=\exp\frac{v_{c}}{RT}[(a-p)(1-\frac{a+2p}{3K_{c}})+\frac{1}{2}\frac{{\left(a-p\right)}^{2}}{E_{c}}]$$

(11) $$\varepsilon_{F}=\exp\frac{v_{F}}{RT}[(a-p)(1-\frac{a+2p}{3K_{F}})+\frac{1}{2}\frac{{\left(a-p\right)}^{2}}{E_{F}}]$$

$v_{c},v_{F}$ are the mole volumes for cement clinker and fly-ash particles; $K_{c},K_{F}$ are the bulk modulus of cement clinker and fly-ash particles; $E_{c},E_{F}$ are the Young’s modulus of cement clinker and fly-ash particles.

Then, the volume of each phase at time t can be expressed as

(12) $$v_{Clin\ker r}(t)=\frac{C_{0}(1-a(t))}{\rho_{Clin\ker}}$$

(13) $$v_{Fa}(t)=\frac{P(1-a_{Fa}(t))}{\rho_{Fa}}$$

(14) $$v_{Hyd}(t)=\frac{k_{1}C_{0}a(t)+k_{2}Pa_{Fa}(t)}{\rho_{Clin\ker}}$$

(15) $$v_{W}(t)=\frac{W_{0}-n_{1}C_{0}a(t)-n_{2}Pa_{Fa}(t)}{\rho_{W}}$$

where $k_{1},k_{2}$ are the volumes of product from unit reactant in hydration and pozzolanic reaction, and $n_{1},n_{2}$ are the volumes of consumed water from the unit reactant in hydration and pozzolanic reaction. Values of these parameters are listed in Bentz’s work [26].

#### 2.1.3. Time-Dependent Development of the Elastic Modulus of Fly-Ash Concrete

For isotropic spherical phases, the self-consistent estimates of the homogenized bulk modulus $K_{pst}$ and shear modulus $\mu_{pst}$ of the binder can be expressed as [20]

(16) $$K_{pst}=\sum_{p}f_{p}K_{p}{\left[1+\alpha (\frac{K_{p}}{K_{pst}}-1)\right]}^{-1}{\left\{{\sum_{q}f_{q}[1+\alpha (\frac{K_{q}}{K_{pst}}-1)]}^{-1}\right\}}^{-1}$$

(17) $$\mu_{pst}=\sum_{p}f_{p}\mu_{p}{\left[1+\beta (\frac{\mu_{p}}{\mu_{pst}}-1)\right]}^{-1}{\left\{{\sum_{q}f_{q}[1+\beta (\frac{\mu_{q}}{\mu_{pst}}-1)]}^{-1}\right\}}^{-1}$$

where $f_{p}$, $f_{q}$ are the volume fractions of each phase, $K_{p}$, $\mu_{p}$ are the bulk and shear modulus of each phase. For spherical inclusions, the Hill tensor α, β is given as follows

(18) $$\alpha =\frac{3K_{pst}}{3K_{pst}+4\mu_{pst}}$$

(19) $$\beta =\frac{6(K_{pst}+2\mu_{pst})}{5(3K_{pst}+4\mu_{pst})}$$

The Mori–Tanaka estimate of the bulk modulus $K_{c}$ and shear modulus $\mu_{c}$ of the binder-aggregate RVE can be expressed in relation to the bulk modulus $K_{a}$, shear modulus $\mu_{a}$, and volume fraction *g* of aggregate [27]

(20) $$\frac{K_{c}}{K_{pst}}=1+\frac{g(K_{a}-K_{pst})}{K_{pst}+a(1-g)(K_{a}-K_{pst})}$$

(21) $$\frac{\mu_{c}}{\mu_{pst}}=1+\frac{g(\mu_{a}-\mu_{pst})}{\mu_{pst}+a(1-g)(\mu_{a}-\mu_{pst})}$$

Aggregate concentration rate *g* can be expressed as the ratio of the aggregate’s volume to the sum of all phases

(22) $$g=\frac{V_{a}}{V_{agg}+V_{c}+V_{fa}+V_{w}+V_{hyd}}$$

Then, the elastic modulus of loaded fly-ash concrete can be quantified:

(23) $$E_{C}=\frac{9K_{c}\mu_{c}}{3K_{c}+\mu_{c}}$$

### 2.2. Materials

The Ordinary Portland Cement (OPC) used in this experiment is of type P.O 42.5, characterized by a density of 3100 kg/m^3^, a Blaine fineness of 300 m^2^/kg, and a 28-day compressive strength of 60.6 MPa. The mineral components of the cement are listed in Table 1 [28].

The chemical components of fly-ash are listed in Table 2.

Grading of macadam is continuous with its diameters lying between 5 and 25 mm, and diameters of medium sand range from 0.8 to 2 mm.

### 2.3. Experimental Program and Testing Procedures

#### 2.3.1. Specimen Preparation and Curing Conditions

The concrete specimens were prism blocks with dimensions of 100 mm × 100 mm × 300 mm, cast in steel molds. All specimens were demolded 24 h after casting. To ensure consistent hydration similar to standard quality control practices, the specimens were cured in a standard curing room at a temperature of 20 ± 2 °C and a relative humidity (RH) of above 95% for the first 28 days. After the 28-day standard curing period, the specimens designated for sustained loading tests were moved to a controlled laboratory environment (Temperature: 20 ± 3 °C; RH: 60 ± 5%) for the remaining duration of the 180-day test period. This unsealed condition allows for moisture exchange with the environment, simulating realistic service conditions.

#### 2.3.2. Compressive Strength Basis and Loading Levels

The sustained load levels (0.3 and 0.5 stress ratios) were determined based on the 28-day axial compressive strength of the prisms. The compressive strength tests were conducted in strict accordance with the Chinese National Standard GB/T 50081-2002 [29]. The measured mean 28-day prismatic compressive strengths were 54.56 MPa for the mixture with 20% fly-ash replacement; 42.00 MPa for the mixture with 40% fly-ash replacement. The number of samples is 3.

These strength characteristics serve as the baseline for determining the applied sustained loads. The methodology of characterizing the mechanical performance of sustainable concrete composites via standard prismatic tests aligns with recent studies on supplementary cementitious materials, such as those by Imoh et al., which emphasize the importance of establishing accurate strength baselines for evaluating long-term property evolution [12].

#### 2.3.3. Elastic Modulus Measurement Protocol

**Figure 2:**
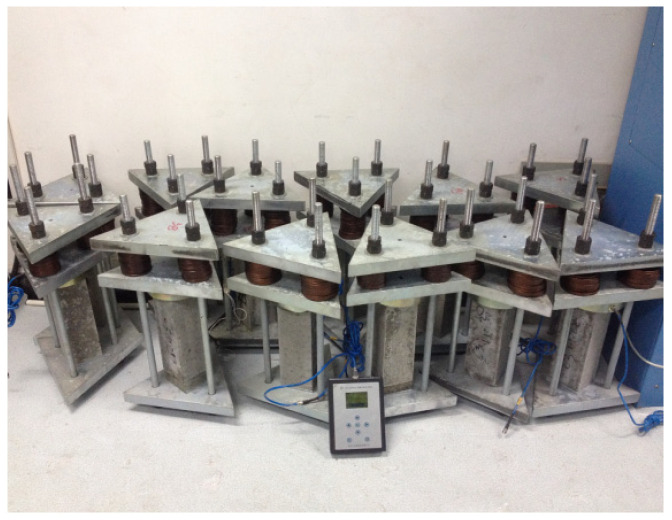
Loading device.

The elastic modulus reported in this study is the static compressive secant modulus, measured in accordance with GB/T 50081-2002. The testing procedure for the sustained-load specimens was as follows:

(I) Unloading: Prior to the modulus test at each designated age, the sustained load was removed, and the specimen was taken out of the spring loading device.

(II) Instrumentation: The loading device is made of hot-galvanized high-strength steel. The loading method is triangular to eliminate eccentricity, and disc springs were installed at each vertex to control the stress level. The device is shown in Figure 2.

The specimen was placed on a hydraulic testing machine. Micro-deformation dial gauges were installed on opposite sides of the specimen with a gauge length of 150 mm to measure the axial deformation.

(III) Loading Protocol: The test involved a pre-loading cycle followed by formal loading. The load was applied continuously at a rate of 0.5 MPa/s.

#### 2.3.4. Sustained Load Application and Monitoring

The sustained load was applied using a self-fabricated spring-reaction device consisting of four high-strength screw rods and stiff springs. The load was initially applied using a hydraulic jack and locked by tightening the nuts against the springs. To address the potential loss of prestress due to concrete creep and spring relaxation, the load magnitude was monitored weekly using a load cell. Any observed deviation exceeding 2% of the target load was immediately corrected by re-tightening the nuts to maintain a constant stress level throughout the 180-day period.

#### 2.3.5. Experimental Design

Two mixtures are designed as listed in Table 3. The fly-ash replacement ratios for A and B are 20% and 40%.

The test programs are illustrated in Table 4. A and B are subgrouped into a non-loaded group (A1,B1) and a loaded group (A2,B2). For A1 and B1, data of their elastic modulus are collected on the 7, 28, 60, 90, 120, and 180 days. Sustained load (20% of the 28-day compressive strength) is applied on specimens of A2 and B2 on the 28th day, and their elastic moduli are tested on the 60, 90, 120 and 180 days. Test data of each group at one test age include the elastic moduli of 3 specimens.

## 3. Results and Discussion

### 3.1. Experimental Results

**Figure 3:**
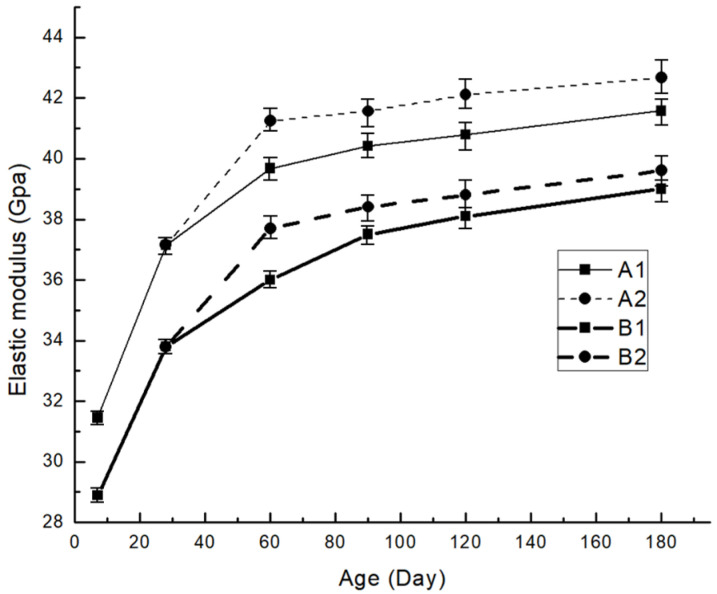
Development of elastic modulus.

Test results are shown in Table 5 and Figure 3. The elastic modulus of A1 is higher than that of B1 at all test ages. The elastic modulus of A1 showed a greater growth rate at early ages, when compared to its 7-day elastic modulus; the 28-day and 60-day elastic modulus increased by 18.09% and 26.13%, while those increasing rates for B1 are 17.04% and 24.61%, respectively. However, the growth rates for the elastic modulus of B1 caught up at later ages. Compared to the 7-day elastic modulus, its 90-day, 120-day, and 180-day elastic modulus increased by 29.66%, 31.73%, and 35.05%, while corresponding rates for A1 are 28.45%, 29.62%, and 32.13%, respectively.

To strictly verify whether the observed increase in elastic modulus is caused by the sustained load rather than random experimental error, a statistical significance test (independent samples *t*-test) was conducted. Specifically, the loaded and unloaded groups of the 20% fly-ash mixture at ages of 60, 90, 120, and 180 days were selected for analysis. The calculated p-value was 0.043, which is less than the significance level of 0.05 (p<0.05). This statistical result confirms that there is a significant difference between the loaded and unloaded groups, providing strong evidence that the sustained load induces a genuine enhancement in the elastic modulus of fly-ash concrete over time.

Fly-Ash concrete with a higher replacement ratio exhibits a lower early-age elastic modulus, which can be ascribed to a lower cement-to-water ratio. Moreover, reaction degrees of fly-ash are about only 10% in concrete in which the fly-ash replacement ratio ranges from 25% to 55%, and about 80% of fly-ash still remains unreacted at 90 days [30]. For a fixed water-to-binder ratio, pastes with higher fly-ash replacements have a lower elastic modulus due to a smaller amount of CSH gel formed in both cement hydration and pozzolanic reaction, but a greater gain in elasticity at later ages as pozzolanic reaction accelerates.

At the same testing age, the elastic moduli of the loaded groups have a small number of increments compared to those of the unloaded. For A2, the increments are 1.56 Gpa, 1.15 Gpa, 1.32 Gpa, and 1.10 Gpa on 60 days, 90 days, 120 days, and 180 days, while those for the B2 group are 1.71 Gpa, 0.91 Gpa, 0.78 Gpa, and 0.56 Gpa. It is observed that this extra gain in elasticity reaches its peak before 90 days, dropping slightly and maintaining steady thereafter. This could be explained by the fact that the extra hydration induced by load is more evident in younger concretes [4,5,31], the ensuing decrease in increment manifests the deceleration of the ‘forced hydration’.

### 3.2. Model Validation

#### 3.2.1. Model Assumptions and Scope

Two key simplifications were adopted to ensure model tractability. First, the assumption of normal stress acting on reacting surfaces is an effective phenomenological approach for the uniaxial compressive loads considered in this study, capturing the dominant thermodynamic driving force. Second, merging water and air into a single zero-stiffness pore phase is justified by the unsealed curing condition (RH 60%), where moisture loss occurs, and the bulk modulus of the remaining fluid is negligible compared to the solid matrix. Consequently, the proposed model is strictly valid for fly-ash concrete under pre-peak uniaxial compression in drying environments

#### 3.2.2. Model Inputs and Calibration Strategy

To ensure the model’s predictive capability and avoid circular fitting, the input parameters are strictly classified into literature-derived fixed values and experimentally calibrated variables.

(I) Fixed Model Inputs

The elastic moduli (e) and Poisson’s ratios (v) of the heterogeneous phases (anhydrous clinker, C-S-H, CH, fly-ash, water, and air) are intrinsic material properties. These values are adopted directly from the nano-indentation results reported by Constantinides and Ulm [22] and are treated as fixed constants throughout the modeling process. The time-dependent degree of hydration (α(t)) and pozzolanic reaction ($\alpha_{p}(t)$) under stress-free conditions are calculated based on the specific chemical composition of the cement and fly-ash used in this study (Table 1), employing the theoretical kinetic models proposed by Han [14]. These kinetic parameters are determined solely by material composition and are independent of the mechanical test data.

(II) Calibration of Load Factors

The model introduces load acceleration factors (ϵ) to quantify the effect of sustained stress on reaction kinetics. These are the only tunable parameters in the framework. The acceleration factors were calibrated using the experimental elastic modulus evolution data of the 20% fly-ash mixture under sustained load.

(III) Validation Approach

To validate the model, the acceleration factors calibrated from the 20% fly-ash mixture group were applied to predict the modulus development of the 40% fly-ash mixture without further adjustment.

#### 3.2.3. Validation Results

Related parameters for the calculation of acceleration factors $\varepsilon_{c}$ and $\varepsilon_{F}$ are listed in Table 6. Detailed parameters of the shrinking core model are taken from Wang and Park’s work [21].

**Figure 4:**
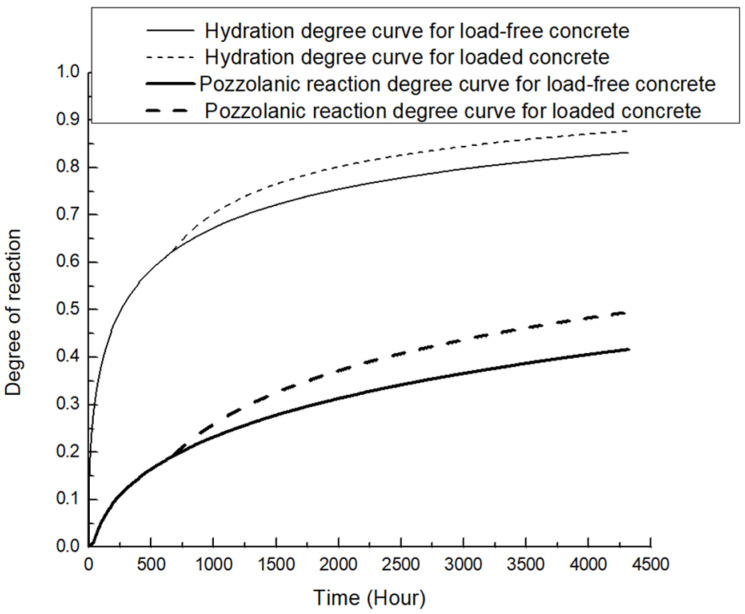
Time-dependent reaction curve for 20% replacement fly-ash concrete.

**Figure 5:**
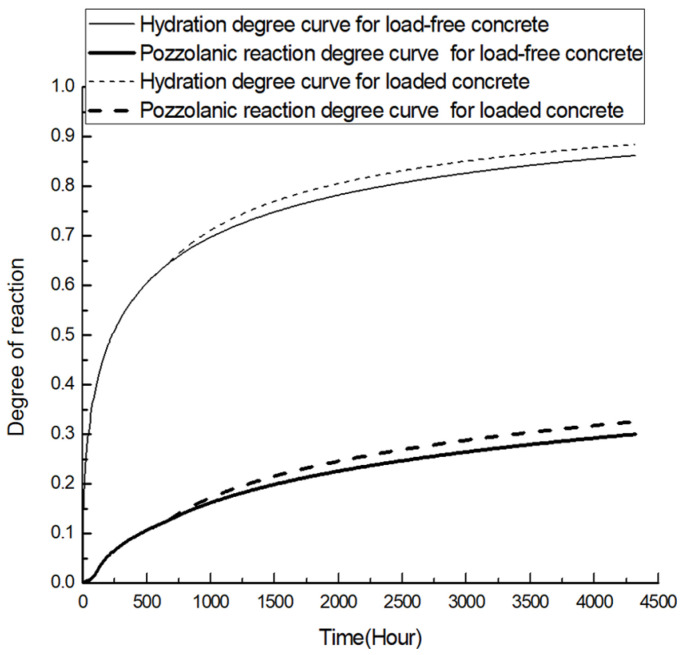
Time-dependent reaction curve for 40% replacement fly-ash concrete.

Time-dependent reaction curves for 20% and 40% replacement fly-ash concrete are presented in Figure 4 and Figure 5, respectively. For both groups of specimens, extra gains in hydration and pozzolanic reaction degree are observed after the imposition of sustained load. It is also observed that the extra reaction is more evident for the 20% replacement.

**Figure 6:**
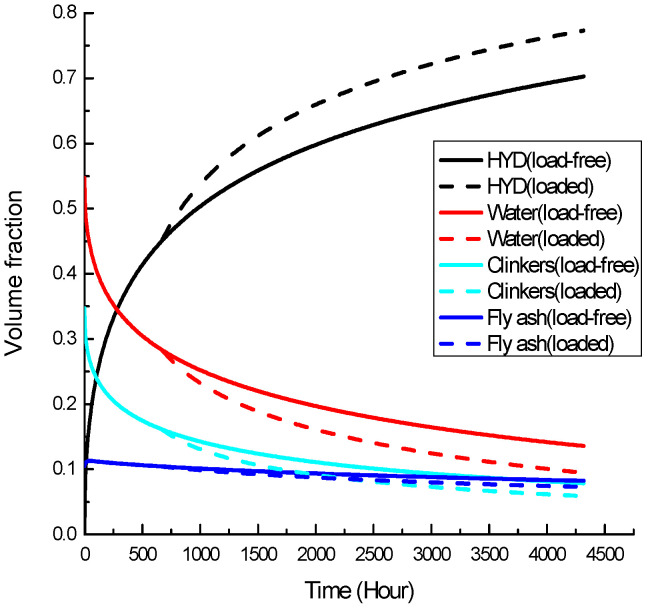
Time-dependent volume fraction of 20% replacement fly-ash concrete.

**Figure 7:**
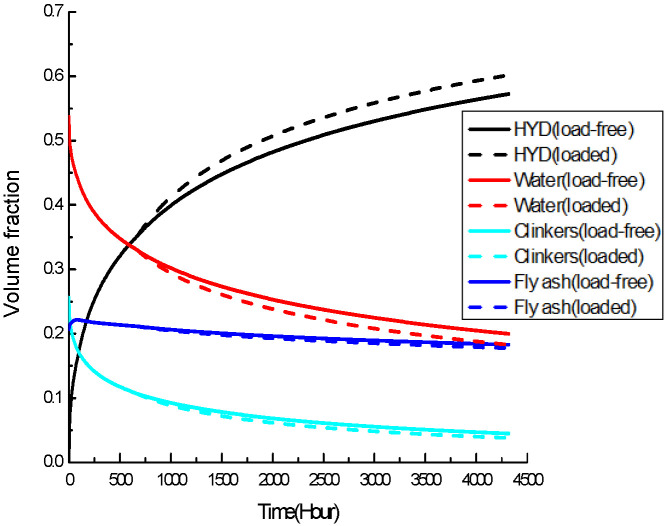
Time-dependent volume fraction of 40% replacement fly-ash concrete.

Time-dependent volume fraction curves of each phase in the binder RVE for both groups of specimens are obtained as shown in Figure 6 and Figure 7. Extra reactions in hydration and pozzolanic reaction have produced a binder with a higher percentage of hydrates and a lower percentage of clinker, fly-ash particles, and water.

Then, the elastic modulus of binder and concrete can be quantified using the Equations (16)–(23). Physical properties of the non-aging phases are listed in Table 7.

**Figure 8:**
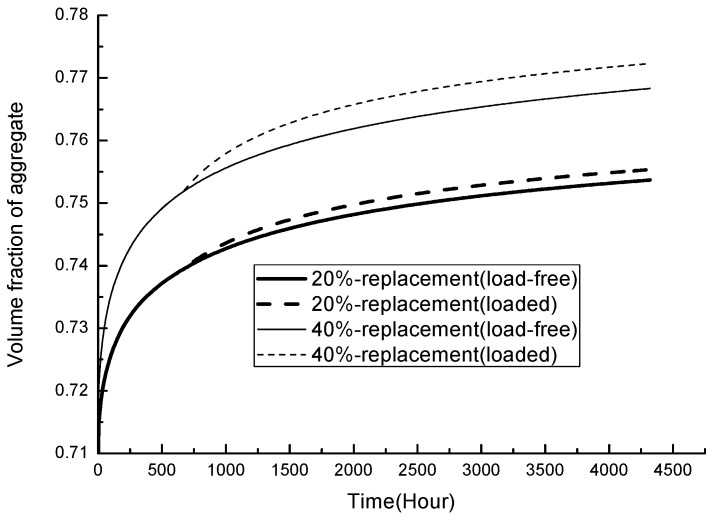
Time-dependent aggregate concentration.

The time-dependent aggregate concentration of loaded concrete has an increment compared to its load-free counterparts; its variation with time for both types of fly-ash concrete is presented in Figure 8.

**Figure 9:**
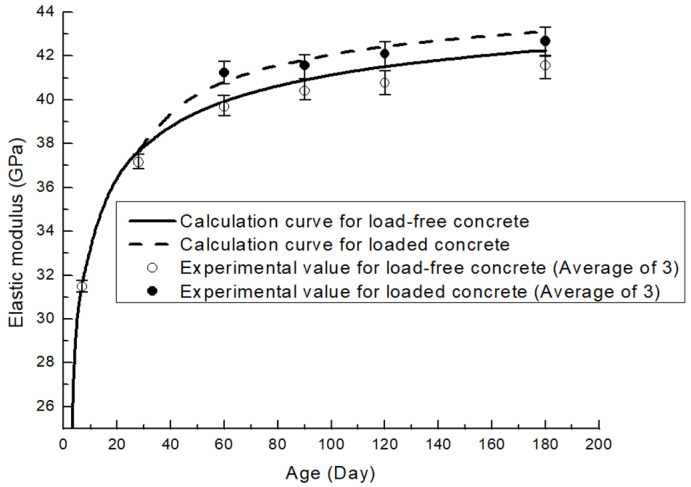
Time-dependent development of 20% replacement FA concrete.

**Figure 10:**
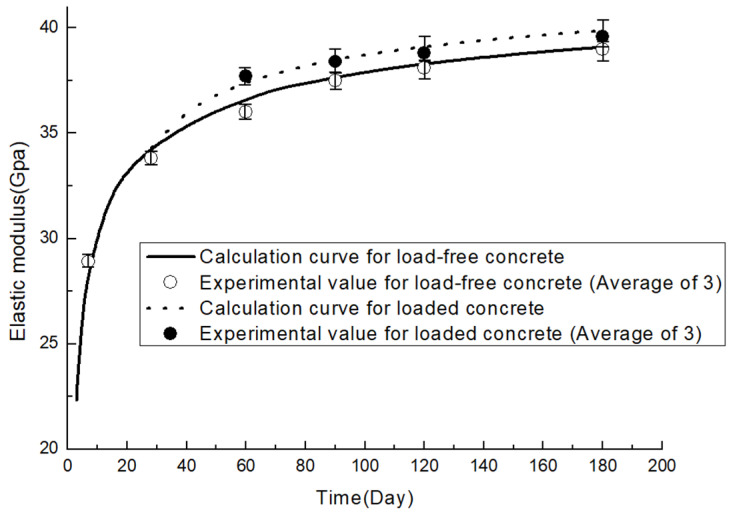
Time-dependent development of 40% replacement FA concrete.

Finally, the time-dependent elastic moduli are given in Figure 9 and Figure 10. Model verification is shown in Table 8. Calculated values are well in line with experimental data, with maximum deviation found to be less than 2%.

### 3.3. Parameter Analysis

#### 3.3.1. Initial Loading Age

**Figure 11:**
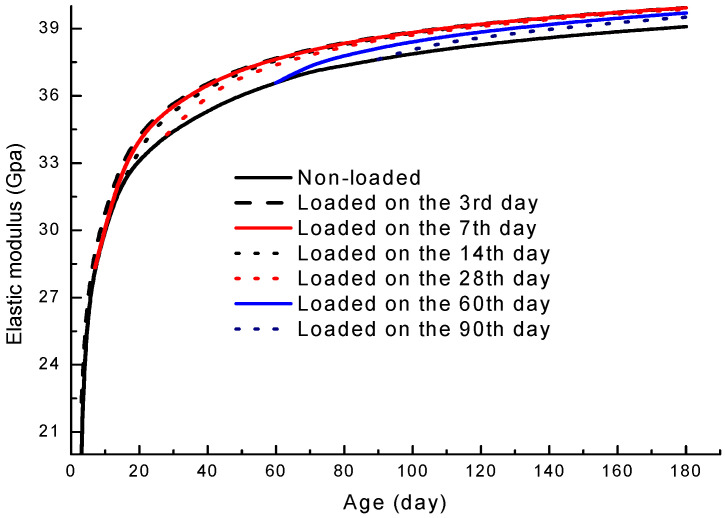
Development of elastic modulus for fly-ash concrete loaded at different ages.

Initial loading age is a crucial parameter in determining the development of elasticity. A simulation was conducted on 40% replacement fly-ash concrete loaded at different ages. To ensure a single variable, 20% of its 28-day prism compressive strength was imposed on specimens on the 3rd, 7th, 14th, 28th, 60th, and 90th day; their development curves are shown in Figure 11.

It can be summarized from the simulation that an earlier initial loading age leads to a higher elastic modulus. Concrete loaded at earlier ages has a larger extra increase in elastic modulus after the action of loading, and it takes a shorter period of time before it reaches a steady increasing rate. This is in line with the work conducted by Sell [4] and Rüsch [5].

For concrete loaded at early ages (the 3rd, 7th, 14th, and 28th days), although the act of loading changes their development curves of elasticity, they tend to have identical elasticity at later ages, with a small discrepancy of less than 0.1 Gpa on the 180th day. This can be explained by the fact that the reaction degree of cement hydration and pozzolanic reaction is controlled by the collaborative effect of age, temperature, and load duration. That the strength and elasticity of loaded concrete at a certain temperature are not sensitive to initial loading ages is in accordance with the test data of Coutinho’s work [10]. However, there are distinguishable decreases in the elasticity increment for concrete loaded at later ages (the 60th and 90th day) when hydration and pozzolanic reactions slow down, and the effect of loading is smaller.

#### 3.3.2. Loading Level

**Figure 12:**
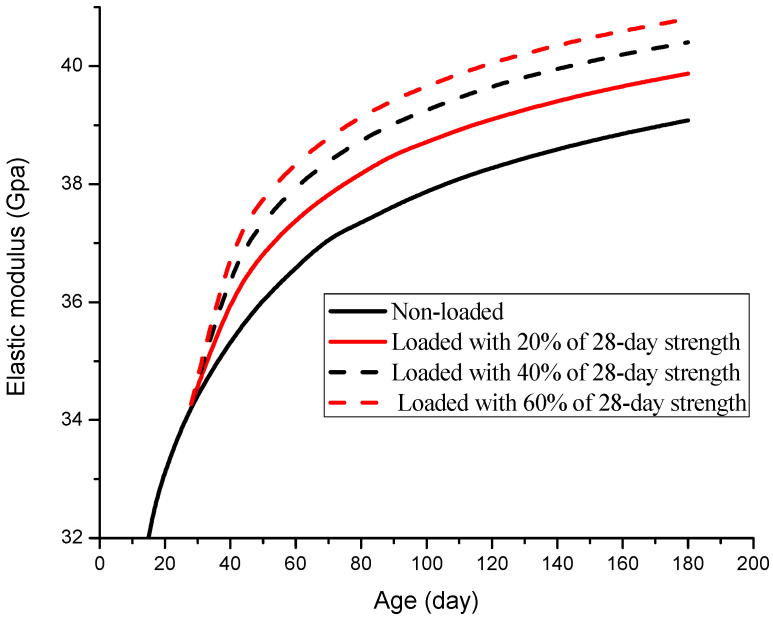
Development of elastic modulus for fly-ash concrete with different loading levels.

The curves of elastic modulus are shown in Figure 12 for the 40% replacement fly-ash concrete loaded with 20%, 40%, and 60% of its 28-day prism compressive strength on the 28th day.

It is obvious that concrete subject to higher stress levels has a greater elastic modulus at the same age. Extra gain in elastic modulus decreases with the loading level increasing because of its non-linear relationship with the extra hydration rate. Actually, there exists a certain point beyond which the damage of concrete induced by a high-stress level will offset the strength and elasticity gain. Shah has found that cement paste under a high-stress level (70% of ultimate compressive stress) has a higher compressive strength than its corresponding concrete of the same age; furthermore, the volume of cement paste was found to decrease while that of concrete increased, which indicates the strengthening and damaging effects coexist in concrete under a high-stress level [8]. Coutinio assumed that the threshold is 0.95 of the ultimate compressive stress when environmental humidity is stable, and strength no longer increases [10]. Price assumed the threshold to be 0.7 of the ultimate compressive stress by calibration [2].

## 4. Conclusions

This study focuses on the elastic modulus of fly-ash concrete under sustained load. Experiments have confirmed the extra gain in loaded fly-ash concrete. The mechanism of this extra increment was analyzed, and the effects of extra hydration and modified aggregate concentration were incorporated into the calculation model.

(I) Test results have shown a small extra gain in elastic modulus for loaded concrete of up to 5% of the non-loaded concrete.
(II) The established model has been proven to be suitable for predicting the elastic modulus of concrete under sustained load.
(III) Parameter analyses based on the model show that concrete loaded at earlier ages tends to have a higher elastic modulus than its counterparts at later ages; moreover, concrete with a higher load level has a larger extra elastic modulus gain.

## Figures and Tables

**Table 1:** Mineral components of OPC (%).

C_3_S	C_2_S	C_3_A	C_4_AF
54.7	19.2	7.1	14.0

**Table 2:** The chemical components of fly-ash (%).

CaO	SiO_2_	AI_2_O_3_	Fe_2_O_3_	MgO	Na_2_O	K_2_O	MnO	TiO_2_	P_2_O_5_	SO_3_	L.O.L
4.35	44.26	33.12	4.39	0.78	0.45	1.71	0.05	1.41	0.73	0.31	4.08

**Table 3:** Mix design.

Type	Cement (kg)	Sand (kg)	Detritus (kg)	Water (kg)	Fly-Ash (kg)	Superplastizer (kg)
A	304	729	1189	152	76	3.8
B	228	729	1189	152	152	3.8

**Table 4:** Specimen design and testing programs.

Specimen Group	Fly-Ash Replacement	Number of Specimens	Testing Age (Day)
A1	20%	18	7, 28, 60, 90, 120, 180
B1	40%	18	7, 28, 60, 90, 120, 180
A2	20%	12	60, 90, 120, 180
B2	40%	12	60, 90, 120, 180

**Table 5:** Elastic modulus at different test ages.

Group	Fly-Ash Replacement	Testing Age (Day)	Elastic Modulus (Average of 3, GPa)	Standard Deviation (GPa)
A1	20%	7	31.46	0.44
		28	37.15	0.52
		60	39.68	0.58
		90	40.41	0.75
		120	40.78	0.79
		180	41.57	0.84
B1	40%	7	28.93	0.43
		28	33.86	0.47
		60	36.05	0.52
		90	37.51	0.61
		120	38.11	0.66
		180	39.07	0.68
A2	20%	60	41.24	0.72
		90	41.56	0.88
		120	42.10	0.93
		180	42.67	1.05
B2	40%	60	37.76	0.69
		90	38.42	0.82
		120	38.89	0.87
		180	39.63	0.96

**Table 6:** Parameters for $\varepsilon_{c}$ and $\varepsilon_{F}$.

$v_{0}$ (cm^3^/mol)	$v_{F}$ (cm^3^/mol)	$a_{40\%}$ (MPa)	$a_{20\%}$ (MPa)	p (MPa)	T (K)
73.2	67.5	10.4	13.2	0.1	297
$K_{c}$ **(GPa)**	$K_{F}$ **(GPa)**	$\mu_{c}$ **(GPa)**	$\mu_{F}$ **(GPa)**	R **(cm^3^ Mpa K^−1^ mol^−1^)**	
116.7	48.9	53.8	35.15	8.314	

**Table 7:** Parameters in the calculation of elastic modulus.

Phase	Bulk Modulus (GPa)	Shear Modulus (GPa)
Clinker [32]	116.7	53.8
Fly-Ash [33]	48.9	35.15
Hydrates [34]	14.3	8.7

**Table 8:** Model verification.

Group	Testing Date (Day)	Experimental Data (GPa)	Model Calculation (GPa)	Error (%)
A1	7	31.46	31.89	1.37
	28	37.15	37.71	1.51
	60	39.68	39.93	0.63
	90	40.41	40.90	1.21
	120	40.78	41.50	1.77
	180	41.57	42.26	1.66
B1	7	28.93	28.52	−1.31
	28	33.86	34.27	1.39
	60	36.05	36.58	1.61
	90	37.51	37.63	0.35
	120	38.11	38.28	0.47
	180	39.07	39.08	0.21
A2	60	41.24	40.83	−0.99
	90	41.56	41.79	0.55
	120	42.10	42.42	−0.82
	180	42.67	43.10	−1.10
B2	60	37.76	37.40	−0.79
	90	38.42	38.50	0.26
	120	38.89	39.11	0.79
	180	39.63	39.87	0.68

## Data Availability

The original contributions presented in this study are included in the article. Further inquiries can be directed to the corresponding author.
